# *LaDAL1* Coordinates Age and Environmental Signals in the Life Cycle of *Larix kaempferi*

**DOI:** 10.3390/ijms24010426

**Published:** 2022-12-27

**Authors:** Xiang-Yi Li, Zha-Long Ye, Dong-Xia Cheng, Qiao-Lu Zang, Li-Wang Qi, Wan-Feng Li

**Affiliations:** State Key Laboratory of Tree Genetics and Breeding, Key Laboratory of Tree Breeding and Cultivation, National Forestry and Grassland Administration, Research Institute of Forestry, Chinese Academy of Forestry, Beijing 100091, China

**Keywords:** age, larch, life cycle, MADS, perennial, transgeneration

## Abstract

Perennial woody plants are long-lived, and their life-cycle events occur in order in each generation, but what drives the occurrence and restart of these events in their offspring is unknown. Based on its age-dependent expression pattern and function, *Larix kaempferi DEFICIENS-AGAMOUS-LIKE 1* (*LaDAL1*), a MADS transcription factor has been suggested to be a time recorder and life-cycle event coordinator. Here, we studied the dynamic spatiotemporal expression pattern of *LaDAL1* in the life cycle of *L. kaempferi* to analyze the molecular mechanism of life-cycle progression. In full view of the life cycle, *LaDAL1* transcription was related with life-cycle progression, and its transcript level increased sharply from age 3 to 5 years, which might be the molecular characteristic of the vegetative phase change, and then stayed at a high level. During sexual reproduction, *LaDAL1* transcript level decreased sequentially during meiosis and embryogenesis, suggesting that meiosis rapidly lowers the age signal, and after fertilization, the age signal was reset to “0” with the embryogenesis. When a seed germinates, the next generation restarts, and the age is re-counted. Altogether, these results not only provide important and novel insights into the life-cycle progression and transgeneration in perennial woody plants, but also advance our understanding of age recording.

## 1. Introduction

The life cycle of a plant is composed of a series of biological events, such as seed germination, flowering, pollination, fertilization, and seed formation. All these events occur in the proper sequence. Most conifers are long-lived and have a long vegetative phase in their life cycle; that is to say, many years are required from seed germination to "flowering". For *Larix kaempferi*, the vegetative phase is ~10 years, after which seed production begins. Furthermore, in the life cycles of temperate-zone conifers, the dormancy-growth cycle occurs during a year. After the first seed production, the conifer continues vegetative growth and produces seeds year after year until it dies. What drives the occurrence and restarting of these events in its offspring is unknown.

In a previous study, we identified 27 age-related transcripts that differ between active juvenile vegetative (1 and 2 years old) and adult reproductive (25 and 50 years old) *L*. *kaempferi* trees [1]. After sequence analysis and cloning, we detected the expression patterns of 20 of these transcripts in active *L*. *kaempferi* trees aged 1 to 13 years, spanning the juvenile vegetative, adult vegetative, and adult reproductive phases [2]. The results showed that six genes have regular expression patterns. *L*. *kaempferi AGAMOUS-Like 2-1* (*LaAGL2-1*)/*DEFICIENS-AGAMOUS-LIKE 1* (*LaDAL1*), *LaAGL2-2*, *LaAGL2-3*, and *L*. *kaempferi SUPPRESSOR OF OVEREXPRESSION OF CONSTANS 1-1* (*LaSOC1-1*) show increased transcription at 5 years, *LaAGL11* shows increased transcription at 3 years, while *L*. *kaempferi APETALA 2-2* (*LaAP2-2*) shows decreased transcription at 1 year [2]. Importantly, over-expression of *LaDAL1* promotes seed germination, bolting, flower initiation, and global proliferative arrest in *Arabidopsis thaliana* [3]. These data suggest that the age-related genes might be messengers that signal age and regulate the dynamic occurrence of life-cycle events. However, their functions and expression patterns in the whole life cycle of *L*. *kaempferi* are still unknown. In addition, whether their age-dependent expression patterns persist in the dormant phase remains unknown.

Notably, a homolog of *LaDAL1* in *P*. *abies*, *DAL1*, also shows increased transcription at 3 or 5 years and its over-expression in *A*. *thaliana* results in early flowering [4]. By analyzing the transcriptome of *Pinus koraiensis*, c77350.graph_c2 was found to be the homolog of *LaDAL1* and also shows an age-dependent expression pattern [5]. Recently, the expression pattern of a homolog of *LaDAL1* in *P*. *tabuliformis* (*PtDAL1*) has been revealed by temporal dynamic transcriptome analysis [6]. It was found that the *PtDAL1* transcript level has the highest correlation coefficient with age in *P*. *tabuliformis* and its over-expression in *A. thaliana* also results in early flowering [6].

All the data from *L*. *kaempferi*, *P*. *abies*, *P*. *koraiensis*, and *P*. *tabuliformis* show that the age-related genes regulate life-cycle events at specific stages of the cycle, and studying the timing of the dynamic changes in their transcription and obtaining a full view of their expression patterns in the life cycle is essential for understanding their relationships with life-cycle events.

## 2. Results and Discussion

**Figure 1:**
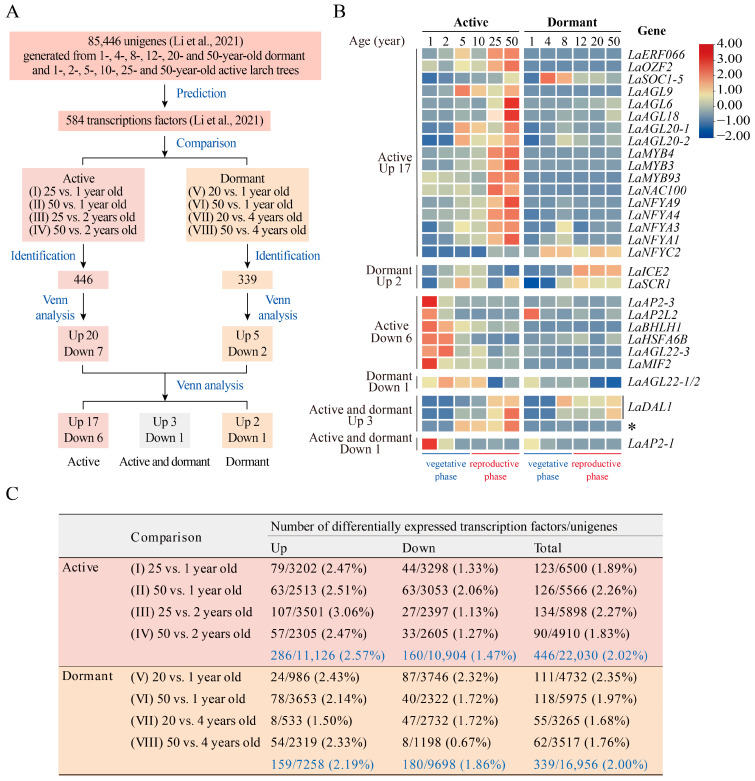
Identification and annotation of 30 differentially expressed transcription factors. (**A**) Analytical flowchart of differentially expressed transcription factors. Branches produced in 2012 were collected from the upper crowns of 1-, 4-, 8-, 12-, 20-, and 50-year-old dormant *Larix kaempferi* trees on 10 March 2013 for transcriptome sequencing [7]. The uppermost main stems produced in the current year had been collected from 1-, 2-, 5-, 10-, 25-, and 50-year-old active *L. kaempferi* trees in July 2011 [8] for transcriptome sequencing [9]. After the removal of buds or needles, the left stems from at least three trees from each age category were pooled and used for RNA extraction. A total of 85,446 assembled unigenes generated from our previous study were used to predict the transcription factors [7]. In the active and dormant phases, the transcript levels of transcription factors in the juvenile vegetative (1-, 2-, and 4-year-old) and adult reproductive (20-, 25- and 50-year-old) phases in *L*. *kaempferi* were compared. (**B**) Heatmap showing the expression patterns of 30 differentially expressed transcription factors assayed by RNA-Seq. Among the 30 transcription factors, the full-length cDNAs of nine were cloned [2,7,10,11] and the sequences of two were almost the same, so the full-length cDNAs of the left 19 were cloned here (Supplemental Table S1). (* transcript not cloned in this study). (**C**) Statistics of differentially expressed transcription factors and unigenes.

Here, we re-analyzed the transcriptomes generated from active and dormant *L. kaempferi* trees [7] (Figure 1A,C), and found that the age-dependent expression pattern of *L. kaempferi DAL1* (*LaDAL1*) remained in the dormant phase (Figure 1B), suggesting that age plays a decisive role in maintaining this expression pattern, and environmental signals that induce, maintain, and release dormancy cannot alter this expression pattern. However, seasonal environmental signals occur periodically over time, raising the questions of what influence they have on the transcription of *LaDAL1*, and how age and seasonal environmental signals are coordinated to maintain the age-dependent expression pattern of *LaDAL1*.

**Figure 2:**
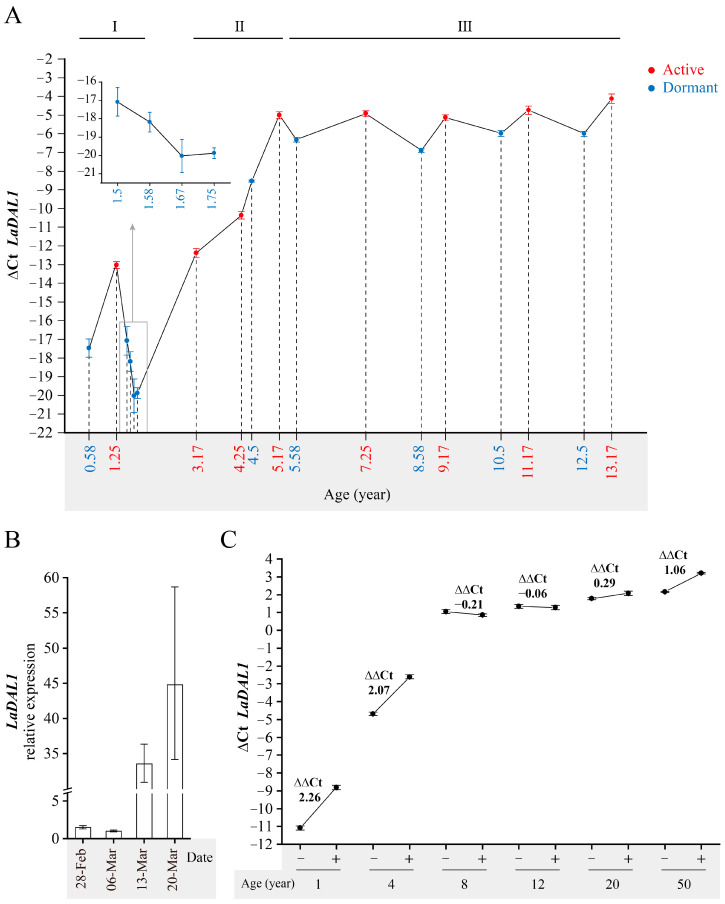
Regulation of *LaDAL1* by age and seasonal environmental signals. (**A**) Expression pattern of *LaDAL1* in the annual growth cycle of *Larix kaempferi* trees of different ages. The main stems or lateral branches from the upper crowns were collected. When sampling, the buds and needles were removed, and the left stems were used for RNA extraction. The tree age was accurately calculated when appropriate. The materials sampled in the active phase are shown by the red color and those sampled in the dormant phase are shown by the blue color, and the detailed sample information was given in the Supplemental Table S2. The *LaDAL1* expression patterns were assayed by qRT-PCR with *L. kaempferi translation elongation factor-1 alpha 1* (*LaEF1A1*) as the internal reference gene. (**B**) Expression pattern of *LaDAL1* during reactivation naturally induced by spring temperature. The main stems of dormant *L*. *kaempferi* trees grown from seeds planted in April 2018 were sampled in early spring of 2019. When sampling, the buds were removed. The *LaDAL1* expression patterns were assayed by qRT-PCR with *L. kaempferi ubiquitin-conjugating enzyme E2 28* (*LaUBC1*) as the internal reference gene. (**C**) Variation of *LaDAL1* transcript levels in dormant *L*. *kaempferi* cuttings after water culture. Branches from 1-, 4-, 8-, 12-, 20- and 50-year-old dormant trees were harvested on 10 March 2013 and then taken to the laboratory for water culture. In each age category, at least 8 branches were sampled as the intact control (“−”), and at least 7 branches were cultured. Following removal of all buds, lanolin was spread over the excised tops of the cuttings and cultured in water with a 16 h photoperiod at 25/20 °C (day/night) and a relative humidity of 75%. Cuttings were sampled after one week (“+”). The *LaDAL1* expression patterns were assayed by qRT-PCR with *LaEF1A1* and *L. kaempferi zinc finger protein* (*LaZFP*) as dual internal reference genes. Error bars represent SD.

To approach these questions, we assessed *LaDAL1* transcription using materials at different phases of the annual growth cycle. Based on the expression pattern of *LaDAL1*, three stages were distinguished (Figure 2A). *LaDAL1* transcript level was low in stage I and high in stage III, and increased continuously in stage II (Figure 2A), again suggesting that age plays a decisive role in maintaining the *LaDAL1* transcript level. In stages I and III, the *LaDAL1* transcript level was high in the active phase and low in the dormant phase (Figure 2A), indicating that environmental signals regulate *LaDAL1* transcription in these two phases. In autumn, environmental signals that induce dormancy decreased *LaDAL1* transcription in stages I and III (Figure 2A), while in spring those that induce reactivation increased its transcription in these stages (Figure 2A,B). Especially in stage I, the *LaDAL1* transcript levels differed during the establishment, maintenance, and release of dormancy (Figure 2A). In stage II, a higher *LaDAL1* transcript level occurred in the dormant phase than in the active phase (Figure 2A), suggesting that *LaDAL1* responds differently to autumn environmental signals that induce dormancy in stages I and III. In conclusion, these results indicate that the regulation of *LaDAL1* by seasonal environmental signals depends on age. Furthermore, the dormant branches sampled from *L. kaempferi* trees of different ages were water-cultured (16 h light, 25 °C). After water culture for one week, more changes in *LaDAL1* transcription occurred in branches from 1-year-old trees (stage I) and 4-year-old trees (stage II) than in those from 8- or 12-year-old trees (stage III) (Figure 2C), further supporting the hypothesis that the environmental regulation of *LaDAL1* is age-dependent. Altogether, after seed germination the seasonal environmental signal and age signal interacted to regulate the *LaDAL1* transcription, resulting in its dynamic temporal expression pattern.

No matter how old the mother tree is, the life cycle restarts after the seeds it produces germinate, and the various life-cycle events of the offspring occur in the same order as in the mother tree. For example, there was no difference, at least in germination and seedling growth, between seeds produced by 90- and 725-year-old *P. nigra* [12]. Here, we hypothesized that the coordinators of life-cycle events are also reset during seed formation and germination. As a time recorder, *LaDAL1* and its homologs also play the role of a life-cycle event coordinator, because their over-expression accelerates the life-cycle progression in *A*. *thaliana* [3,4,6,13]. So how is the *LaDAL1* transcript level reset to “0” in the next generation? As a coordinator of life-cycle events in *A. thaliana*, miR156 is reset during sexual reproduction and embryogenesis, and this is consistent with the life cycle resetting of *A. thaliana* [14].

**Figure 3:**
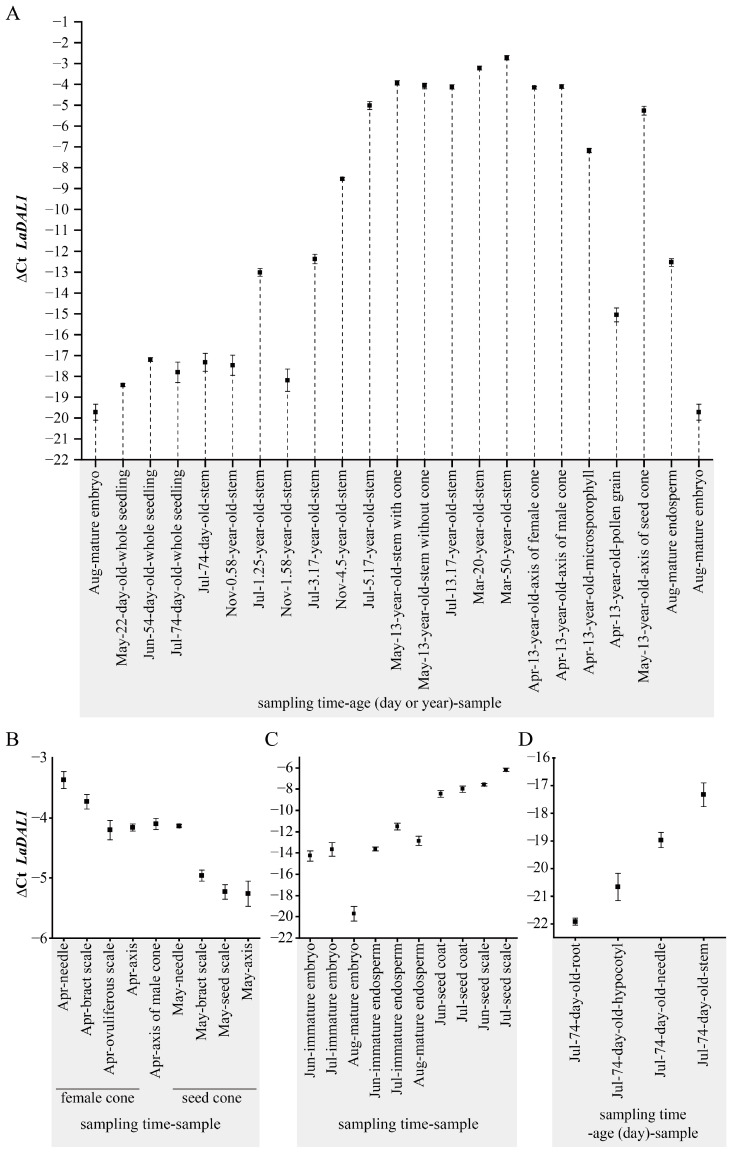
Dynamic spatiotemporal expression pattern of *LaDAL1* in the life cycle of *Larix kaempferi*. (**A**) The *LaDAL1* expression pattern in the *L. kaempferi* life cycle. Samples included the embryo and endosperm of mature seeds (*n* = 10), 22-day-old seedlings (*n* = 10), 54-day-old seedlings (*n* = 10), 74-day-old seedlings (*n* = 6), the uppermost stem from 13-year-old trees with (*n* = 4) or without cones (*n* = 4), the axis of male and female cones and the microsporophylls (*n* ≥ 10), pollen grains (after modulation for 24 h), and the axis of immature seed cones (*n* ≥10). These cones were collected from 13-year-old active *L*. *kaempferi* trees (*n* ≥ 4). The *p*-value was calculated between May-13-year-old-stem with cones and May-13-year-old-stem without cones. One-way ANOVA Duncan’s test was used for statistical analysis. (**B**) Variation of *LaDAL1* transcript levels in different cone tissues. Samples included the ovuliferous scale, needle, bract scale, and axis of female cones (*n* ≥ 10), the axis of male cones and the seed scale, bract scale, needle, and axis of immature seed cones (*n* ≥ 10); these cones were collected from 13-year-old active *L*. *kaempferi* trees (*n* ≥ 4). (**C**) Variation of *LaDAL1* transcript levels in different seed tissues. Embryo and endosperm of mature seeds (*n* = 10) were sampled from the grafted trees planted in 1985 (sampled on 22 August 2020). The embryo, endosperm, and seed coat of immature seeds (*n* = 10) and the seed scale were sampled from 13-year-old (*n* = 4, sampled on 30 June 2022) and the grafted trees planted in 1985 (*n* = 3, sampled on 27 July 2022). (**D**) Variation of *LaDAL1* transcript levels in stem, needle, hypocotyl, and root of 74-day-old seedlings. They were grown from seeds planted on 22 April 2021 and sampled on 4 July 2021 (*n* = 15). All of the above qRT-PCR assays were performed with *LaEF1A1* as the internal reference gene. Error bars represent SD.

In 13-year-old *L. kaempferi* trees, high *LaDAL1* transcript levels were found in the different tissues of female cones, the needle, bract, seed scale and axis of seed cones, and the axis of the male cone (Figure 3A,B), suggesting that *LaDAL1* reset did not occur during the formation of these tissues. The *LaDAL1* transcript level in microsporophyll was ~8 times (2^−∆∆Ct^) lower than that in the axis of the male cone, and it was ~256 times lower in the pollen grain than in the microsporophyll (Figure 3A), indicating that the *LaDAL1* transcript level drops sharply during pollen grain production. Compared with pollen grains, the *LaDAL1* transcript level in mature seed embryos was 32 times lower (Figure 3A), indicating that embryogenesis caused the second sharp decrease. More precisely, it was during the embryo maturation, the second sharp decrease occurred (Figure 3C). With seed germination, the new life cycle restarted, and simultaneously the *LaDAL1* transcript level also began to increase (Figure 3A). These results suggested that meiosis and embryogenesis reduce *LaDAL1* transcription, and *LaDAL1* is also reset during sexual reproduction and embryogenesis.

We also captured the dynamics of time information in other materials. The microsporophyll consists of the sporangium wall (2n) and the microsporangium, where meiosis occurs to produce pollen grains (male gametophytes) (n); the sporangium wall is derived from the mother tree by mitosis. Compared with the axis (2n) of the male cone, the *LaDAL1* transcript level in microsporophyll was lower (Figure 3A), and compared with the microsporophyll, it was lower in the pollen grain (Figure 3A), suggesting that the sporangium wall and axis of the male cone are “older” than the pollen grain. The seed coat (2n) is derived from the mother tree without meiosis, the endosperm (female gametophyte) (n) is produced after meiosis, and the embryo (2n) is produced after fertilization. The *LaDAL1* transcript level in endosperm was lower than that in the seed coat, while it was lower in the newly-formed mature embryo than that in the endosperm (Figure 3A,C), suggesting that the seed coat is “older” than the endosperm and mature embryo. The spatial distribution pattern of miR156 was also revealed in the reproductive organ of *A. thaliana*, showing that the mature miR156 levels are higher in the newly formed tissues (pollen and embryo) than maternal tissues (filament, anther wall, sporangium wall, vascular bundle in the microsporangium, and integument) [14]. The spatial distribution pattern of *LaDAL1* in the reproductive organ of *L. kaempferi* showed the opposite to miR156, because *LaDAL1* transcript level was higher in maternal tissue (seed coat) than in the newly formed tissue (embryo). These results further suggested that meiosis and embryogenesis are key events in the resetting of the life cycle and its coordinators in *A. thaliana* and *L. kaempferi*.

In addition, we found no difference in the *LaDAL1* transcript level in the stems of 13-year-old trees that did or did not produce cones (*p* > 0.05; Figure 3A), suggesting that *LaDAL1* transcription is not directly related to “flowering”. With seed germination, the *LaDAL1* transcript level began to increase (Figure 3A); in 74-day-old seedlings, it was highest in the stem and lowest in the root (Figure 3D), suggesting that the root is younger and more suitable as explants. The data in Figure 3B showed that the *LaDAL1* transcript levels were high in the tissues of male cones, female cones, and seed cones, indicating that these materials were at the end-stage of the life cycle. The *LaDAL1* transcript level increased slightly in 20- and 50-year-old trees (Figure 3A).

**Figure 4:**
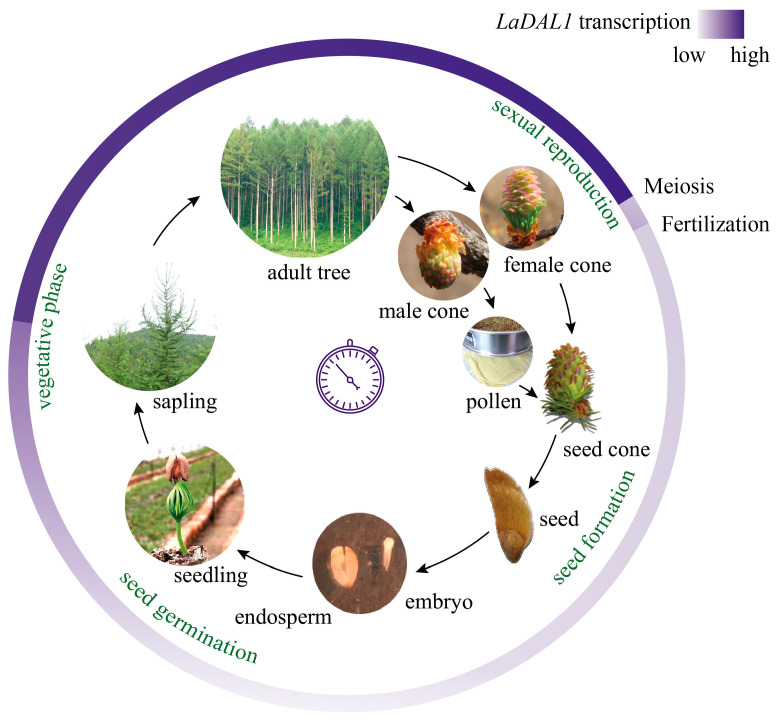
Schematic depicting the life cycle of *Larix kaempferi* and the expression pattern of *LaDAL1*. In this full view of *L. kaempferi* life cycle, the *LaDAL1* transcript level increased from seed germination, then increased sharply in the vegetative phase change, and decreased during meiosis and embryogenesis.

Altogether, we showed the dynamic spatiotemporal expression pattern of *LaDAL1* in the life cycle of *L. kaempferi*, providing clues for analyzing the molecular mechanism of life-cycle progression controlled synergistically by seasonal environmental signals and age. *LaDAL1* transcription was related with the life-cycle progression (Figure 4), and its transcript level increased sharply from age 3 to 5 years, which might be the molecular characteristic of the vegetative phase change, and then stayed at a high level. During sexual reproduction, the *LaDAL1* transcript level decreased sequentially during meiosis and embryogenesis (Figure 4). As a messenger of age signals, *LaDAL1* participates in regulating the *L. kaempferi* life cycle by integrating external environmental signals. Meiosis rapidly lowers this age signal, and embryogenesis completely resets the age signal to “0”. During *A. thaliana* sporogenesis and gametogenesis, compared with microspore and megaspore mother cells, the activity of miR156, another messenger of age signals, increases in the sperm cell and egg cell, respectively [14], also suggesting that meiosis reset the age signal. As a seed germinates, the next generation restarts, and the age is re-counted. Our findings provide new insights into the growth and development of perennial woody plants in the context of their whole life cycles.

## 3. Materials and Methods

### 3.1. Transcriptome Data

The transcriptome data were generated in our previous studies [7,9]. Branches produced in 2012 were collected from the upper crowns of 1-, 4-, 8-, 12-, 20-, and 50-year-old dormant *L. kaempferi* trees on 10 March 2013 for transcriptome sequencing [7]. The uppermost main stems produced in the current year were collected from 1-, 2-, 5-, 10-, 25-, and 50-year-old active *L. kaempferi* trees in July 2011 [8] for transcriptome sequencing [9]. After removal of buds or needles, the samples from at least three trees from each age category were pooled, frozen in liquid nitrogen, and stored at −80 °C until RNA extraction and library construction.

These trees were grown from seeds and located in Dagujia seed orchard (42°22′ N, 124°51′ E), Liaoning Province, Northeast China. About 10 years is required for larch to achieve the capacity to “flower”. We selected these ages because they constitute an entire rotation period (40–50 years) from establishment to harvest and include the vegetative and reproductive phases.

Transcriptome libraries were constructed for each age category in our previous studies [7,9]. A total of 140,691,414 reads were produced from the six age categories of dormant *L. kaempferi* trees [7], and a total of 151,413,654 reads were produced from the six age categories of active trees [9]. All reads were assembled into 85,446 unigenes, from which 584 transcription factors belonging to 50 families were predicted [7].

Transcripts from RNA-Seq data were quantified in our previous study [7]. Twelve sets of sequencing reads were mapped to the assembled reference transcripts using Bowtie [15]. RNA-Seq by expectation maximization [16], an accurate method of transcript quantification from RNA-Seq data, was used to estimate the transcript abundance. The expression of genes was normalized with edgeR (empirical analysis of digital gene expression data in R) [17]. Fragments per kilobase of transcript per million fragments (FPKM) was used to measure the normalized expression value.

### 3.2. Identification of DEUs

The read counts for unigenes were used to identify differentially expressed unigenes (DEUs) with the software GFOLD, which is especially useful when no replicate is available [18]. Eight pair-wise comparisons were applied to identify DEUs. In the active phase there were four comparisons: (I) 25 vs. 1 year old; (II) 50 vs. 1 year old; (III) 25 vs. 2 years old; and (IV) 50 vs. 2 years old, and in the dormant phase, there were another four comparisons: (V) 20 vs. 1 year old; (VI) 50 vs. 1 year old; (VII) 20 vs. 4 years old; and (VIII) 50 vs. 4 years old. After pairwise comparison, DEUs were determined using the GFOLD with |GFOLD value| ≥ 0.5 and |log_2_ (fold change)| ≥ 1. The Venny 2.1 tool was used to find the unigenes shared between different comparisons (http://bioinfogp.cnb.csic.es/tools/venny/index.html, accessed on 15 May 2020). The first Venn analysis was performed to analyze differentially expressed transcription factors identified from active and dormant phases separately, and the second Venn analysis was performed to analyze the first Venn analysis results. Then the TBtools software package (Toolbox for Biologists v1.098661) [19] was used to generate heatmaps from FPKM values normalized by row and to visualize the expression patterns.

### 3.3. Plant Materials

#### 3.3.1. The Materials Used for Determining the Dynamic Temporal Expression Pattern of LaDAL1 in the Annual Growth Cycle of Larix kaempferi Trees of Different Ages

*L. kaempferi* trees at different phases of the annual growth cycle were used for assessing *LaDAL1* transcription (Figure 2). These trees were grown from seeds and of different ages, and their main stems or lateral branches from the upper crowns were sampled. The active phase of *L. kaempferi* tree is from the middle of April to early September in Dagujia seed orchard. When sampling, the buds and needles were removed, and the left stems were used. The trees sampled in Beijing were at the dormant phase from October to January (Figure 2A, extra small graph) and at the transition from dormant to active phase from February to March (Figure 2B). All information about the trees and sampling is given in Supplemental Table S2. The tree age was accurately calculated when appropriate.

#### 3.3.2. The Materials Used for Determining the Dynamic Spatiotemporal Expression Pattern of LaDAL1 during the *L. kaempferi* Life Cycle

To determine the *LaDAL1* expression pattern during the *L*. *kaempferi* life cycle, materials at different stages of the life cycle were used (seeds, seedlings, saplings, mature trees, and cone tissues; details in Supplemental Table S3).

##### Seeds and Seedlings

**Figure 5:**
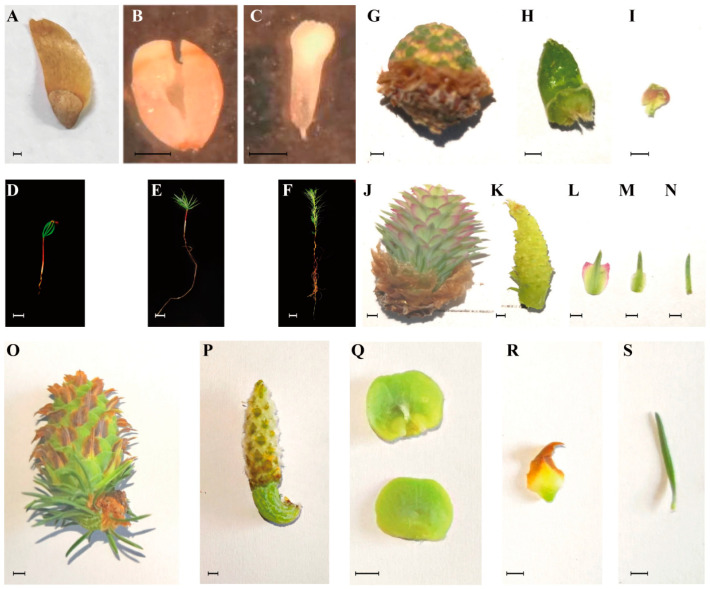
Plant materials used for *LaDAL1* expression pattern analysis during the life cycle of *Larix kaempferi*: (**A**) mature seed, (**B**) endosperm from a mature seed, (**C**) embryo from a mature seed, (**D**) 22-day-old seedling, (**E**) 54-day-old seedling, (**F**) 74-day-old seedling. (**G**) male cone collected on 13 April 2020, (**H**) axis of a male cone, (**I**) microsporophyll, (**J**) female cone collected on 13 April 2020, (**K**) axis of a female cone, (**L**) ovuliferous scale, (**M**) bract scale from a female cone, (**N**) needle from a female cone, (**O**) immature seed cone collected on 14 May 2020, (**P**) axis of a seed cone, (**Q**) seed scales, (**R**) bract scale from a seed cone, (**S**) needle from a seed cone. Black scale bars, 1 mm; white scale bars, 1 cm.

On 22 August 2020, the mature seeds (Figure 5A) were collected from the grafted trees planted in 1985 and stored at 4 °C, and the endosperm (Figure 5B) and embryo (Figure 5C) were collected from ten mature seeds on 3 May 2021 after soaking them in water for two days. The embryo, endosperm, and seed coat of immature seeds (*n* = 10) and the seed scales were sampled from 13-year-old trees (*n* = 4) on 30 June 2022 and the grafted trees planted in 1985 (*n* = 3) on 27 July 2022. The whole seedlings of 22-day-old (*n* = 10) were sampled on 13 May 2021, 54-day-old (*n* = 10) were sampled on 14 June 2021, and 74-day-old (*n* = 6) were sampled on 4 July 2021 (Figure 5D–F). Furthermore, the stems, needles, hypocotyls, and roots were sampled from 74-day-old seedlings (*n* = 15) on 4 July 2021.

##### Saplings and Mature Trees

The stems sampled from 0.58-, 1.25-, 3.17-, 4.5-, 5.17-, 13.17-, 20-, and 50-year-old trees were also used here (Supplemental Tables S2 and S3). The uppermost stems from 13-year-old trees (*n* = 8) were sampled on 14 May 2020, four of which had cones and another four had not, and they were sampled separately.

##### Cones

The male cones (*n* ≥ 10) (Figure 5G) were collected from 13-year-old active *L. kaempferi* trees (*n* ≥ 4) on 13 April 2020, and the axis of male cones (Figure 5H), microsporophylls (Figure 5I), and pollen grains (after modulation for 24 h) were sampled immediately. Before pollination, the female cones (*n* ≥ 10) (Figure 5J) were collected from 13-years-old active *L. kaempferi* trees (*n* ≥ 4) on 13 April 2020, and the axis of female cones (Figure 5K), ovuliferous scales (Figure 5L), bract scales (Figure 5M), and needles (Figure 5N) were sampled immediately. The immature seed cones (Figure 5O) were collected from 13-years-old active *L. kaempferi* trees (*n* ≥ 4) on 14 May 2020 after pollination, and the axis of immature seed cones (Figure 5P), seed scales (Figure 5Q), bract scales (Figure 5R), and needles (Figure 5S) were sampled immediately.

### 3.4. Water Culture Experiments

Branches from 1-, 4-, 8-, 12-, 20- and 50-year-old dormant *L*. *kaempferi* trees were harvested on 10 March 2013 in Dagujia and then taken to Beijing (Supplemental Table S2). At least 8 branches from each age category were sampled as the intact control on 10 March 2013 after removal of all buds. On 13 March 2013 water culture experiments were performed, and at least 7 branches from each age category were cultured. Following removal of all buds, they were treated with lanolin that was spread over the excised tops of the cuttings. A water culture system was set up in a growth chamber with a 16 h photoperiod, temperatures at 25/20 °C (day/night) and a relative humidity of 75%. Branches were sampled after culture for one week.

### 3.5. Sequence Analysis, Full-Length cDNA Cloning, and Annotation

The open reading frame (ORF) finder (https://www.ncbi.nlm.nih.gov/orffinder/, accessed on 4 April 2020) was used to identify the ORFs of the differentially expressed transcription factors. Primers (Supplemental Table S4) were designed to clone their full-length cDNA sequences with Platinum^®^ Taq DNA polymerase (Invitrogen, Carlsbad, CA, USA). The PCR products were purified with a gel extraction kit (Tiangen, Beijing, China), ligated into the pEASY^®^-T1 simple cloning vector (TransGen Biotech, Beijing, China), and sequenced. The deduced amino acids were used for blast analysis with *A. thaliana* homologs, and based on the results, they were designated and annotated again. The full-length cDNA sequences have been submitted to GenBank.

### 3.6. RNA Extraction and cDNA Synthesis

Total RNA was extracted with the EasyPure RNA Kit (TransGen Biotech) according to the manufacturer’s protocol. A 2.5 µg of total RNA was reverse-transcribed into cDNA with the TransScript II One-step gDNA Removal and cDNA Synthesis SuperMix Kit (TransGen Biotech), and subsequently diluted for sequence cloning and quantitative reverse transcription polymerase chain reaction (qRT-PCR).

### 3.7. QRT-PCR

The qRT-PCR analysis was performed on a Bio-Rad CFX96 PCR system, using a TB Green^®^ Premix Ex Taq™ (Tli RNase H Plus) (Takara, Shiga, Japan). Each reaction was carried out on 2 µL of diluted cDNA sample, in a total reaction system of 25 µL. The reaction procedure was set up according to the manufacturer’s protocol: 95 °C for 30 s, then 45 cycles at 95 °C for 5 s, and at 60 °C for 30 s, followed by a melting step from 65 to 95 °C. The primers for qRT-PCR are listed in Supplemental Table S4.

The *L. kaempferi translation elongation factor-1 alpha 1* (*LaEF1A1*) (GenBank accession no. JX157845) [20], *L. kaempferi zinc finger protein* (*LaZFP*) (GenBank accession no. MZ965074), and *L. kaempferi ubiquitin-conjugating enzyme E2 28* (*LaUBC1*) (GenBank accession no. ON887160) were used as the internal reference genes. Dual internal reference genes (*LaEF1A1* and *LaZFP*) were used in the qRT-PCR analysis of water culture experiments.

Relative quantification using △CT values (CT_reference gene_ − CT*_LaDAL1_*), where CT is the threshold cycle, was used to present the transcript level shown in Figure 2A,C and Figure 3. The relative expression ratio was expressed using the 2^−∆∆Ct^ method for the data shown in Figure 2B. The *LaDAL1* transcript level was normalized to the constitutive transcript level of *LaUBC1*, and the sample with the minimum transcript level was used for calibration and was set to a value of 1. The qRT-PCR was performed with three or four technical replicates, and the data are shown as the mean ± SD.

## Data Availability

All materials are available from the corresponding author on request.

## Supplementary Materials

The following supporting information can be downloaded at: https://www.mdpi.com/article/10.3390/ijms24010426/s1.
